# Supplementary material for: Fungal Species Causing Maize Leaf Blight in Different Agro-Ecologies in India
Source: Pathogens. 2021 Dec 14;10(12):1621. doi: 10.3390/pathogens10121621 (PMC8705428; doi:10.3390/pathogens10121621)
Supplement: Supplementary file 1 [file pathogens-10-01621-s001.zip › pathogens-1479455-supplementary.pdf]

**Supplementary:**

**Table S1.** Culture strains, host, country and ITS GenBank accession numbers used as references in this study.

| Species | Strain number | Host | Country | GenBank accession number | |
|---|---|---|---|---|---|
| | | | | ITS | GAPDH |
| *Alternaria alternata* | CBS 916.96 | NA | NA | KF465761 | |
| *Bipolaris cynodontis* | CBS 109894 | *Cynodon dactylon* | Hungary | KJ909767 | |
| *B. eleusines* | CBS 274.91 | *Eleusine indica* | Australia | KJ909768 | |
| *B. maydis* | CBS 137271 | *Zea mays* | USA | AF071325 | KM034846 |
| *B. salviniae* | CBS 308.90 | Not availlable | Australia | MH873897 | |
| *B. sorokiniana* | CBS 110.14 | *Hordeum* sp. | USA | KJ922381 | |
| *B. yamadae* | CBS 202.29 | *Panicum miliaceum* | Japan | KJ909779 | |
| *B. zeae* | BRIP 11512IsoP* | *Zea mays* | Australia | KJ415538 | |
| *B. zeicola* | FIP532 | *Zea mays* | USA | KM230398 | KM034815 |
| *Curvularia hawaiiensis* | CBS 173.57 | *Oryza sativa* | Hawaii | JN601029 | |
| *C. inaequalis* | CBS 102.42 | Sand dune soil | France | KJ922375 | KM061787 |
| *C. lunata* | CBS730.96 | Human lung biopsy | USA | JX256429 | JX276441 |
| *C. papendorfii* | CBS 308.67 | *Acacia karroo* | South Africa | KJ909774 | KM083617 |
| *C. richardiae* | BRIP 4371 | *Richardia brasiliensis* | Australia | KJ415555 | KJ415391 |
| *C. spicifera* | CBS 274.52 | Soil | Spain | JN192387 | JN600979 |
| *C. trifolii* | ICMP6149 | *Setaria glauca* | New Zealand | JX256434 | JX276457 |
| *C. ellisii* | CBS193.62 | Air | Pakistan | JN192375 | |
| *C. graminicola* | BRIP 23186 | *Aristida ingrata* | Australia | JN192376 | JN600964 |
| *C. siddiquii* | CBS 142.78 | Unknown | Egypt | MN688824 | MN688851 |
| *C. siddiquii* | CBS 196.62 | Air | Pakistan | MN688823 | MN688850 |
| *Curvularia* sp. | BRIP 61674 | *Oryza australiensis* | Australia | | KU552661 |

**Table S2.** Distribution of morphological and pathogenic variability in the total population of maize leaf blight isolates sampled from six maize production zones of India.

| S. No | Group | Hotspots included (Identified species*) | Morphological and pathological profiles | Percent composition |
|---|---|---|---|---|
| 1. | Group-1 | *BmPhRj4* (*Bipolaris maydis*), *BmAmRj4* (*Alternaria* sp.) and *BmLdPj2* (*B. maydis*) | Rough colony with no zonation and irregular margins, slow to medium colony growth, mild to moderate virulence on host, conidial dimensions ranging from 9.8 x 3.4 µm to 18.6 x 5.8 µm, 2.5 to 6.0 septate, incubation period 72 h on host and disease severity 1.5 to 4.3 on the rating scale, symptoms ranging from Type-I, -II and -IV | 15+15+34=64 (18.28%) |
| 2. | Group-2 | *BmBjUa1* (*B. maydis*), *BmCgRj4* (*B. maydis*), *BmPtUa1* (*B. maydis*) and *BmGdGj5* (*Carvularia papendorfii*) | Rough colony with no zonation and regular margins, medium to fast colony growth, having moderate virulence on host, conidial dimensions 7.6 x 3.1 to 16.9 x 4.1 µm, 1.3 to 5.8 septate, incubation period 48 to 72 h and disease severity 2.6 to 3.3 on the rating scale, symptoms Type-I and -II | 23+18+18+12=71 (20.28%) |
| 3. | Group-3 | *BmPnDl12* (*B. maydis*) and *BmLhRj4* (*C. sporobolicola*) | Rough colony with zonation and irregular margins, medium colony growth, having moderate to high virulence, conidial dimensions 17.2 x 3.7 to 19.3 x 5.0 µm, 3.7 to 4.8 septate, incubation period 72 h and disease severity of 2.7 to 3.7 on the rating scale, Symptoms Type-III and -IV | 15+11=26 (7.42%) |
| 4. | Group-4 | *BmBsRj4* (*B. maydis*), *BmDnRj4* (*B. maydis*), *BmKgUa1* (*B. maydis*), *BmKrHr2* (*B. maydis*), *BmKtRj4* (*B. maydis*) and *BmSkRj4* (*C. siddiquii*) | Smooth appressed colony with no zonation and regular margins, slow to fast colony growth, having mild to high virulence, conidial dimensions 10.1 x 2.6 to 16.8 x 3.1 µm, 2.5 to 4.7 septate, incubation period 48 to 96 h and disease severity 1.9 to 3.6 on the rating scale. Symptoms Type-1, -II, -III and -V | 11+19+21+20+23+16=110 (31.42%) |

| | | | | |
|---|---|---|---|---|
| 5. | Group-5 | *BmMdKa6* (*B. zeicola*), *BmMyKa6* (*B. maydis*), and *BmSmBh3* (*C. graminicola*-like fungus) | Smooth appressed colony with zonation and regular margins, medium to fast colony growth, conidial dimensions 7.8 x 6.1 to 13.1 x 3.8 µm, 2.6-4.2 septate, having mild to high virulence, 72 to 76 hincubation period and disease severity 1.7 to 4.3 on rating, Symptoms Type-I, -IV and -V | 13+9+18=40 (11.42%) |
| 6. | Group-6 | *BmDhBh3* (*B. maydis*) and *BmAdGj5* (*C. papendorfii*) | Rough appressed colony with no zonation and regular margins, medium colony growth, conidial dimensions 12.1 x 4.3 to 14.4 x 4.5 µm, 2.9 to 3.5 septate, having high virulence, 72 h incubation period and disease rating 3.7 to 4.5 on the rating scale. Symptoms Type-I and -VI | 27+12=39 (11.14%) |

*Respective species are photogenically identified in Figure 1A, B.

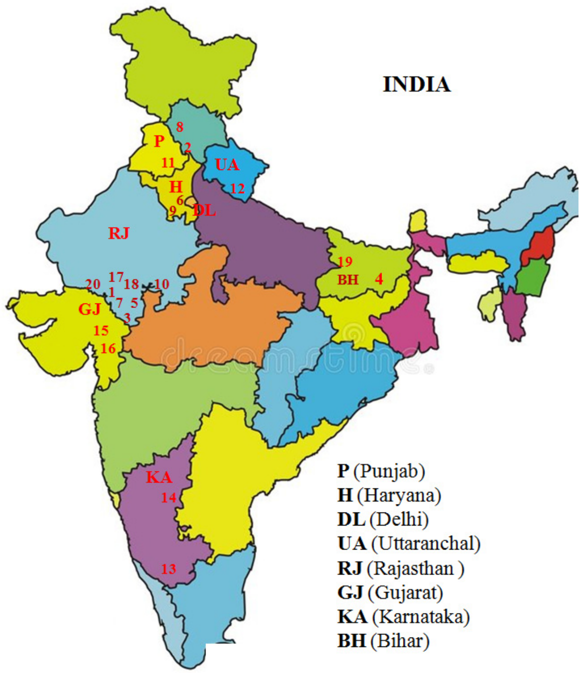


**Figure S1.** Hotspot locations covered under survey and surveillance for monitoring maize leaf blight symptoms: **1** (*Pichola*); **2** (*Bajaura*); **3** (*Banswara*); **4** (*Dholi*); **5** (*Chittorgarh*); **6** (*Patel Nagar*); **7** *(Dungarpur*); **8** (*Kangra*); **9** (*Karnal*); **10** (*Kota*); **11** (*Ludhiana*); **12** (*Pantnagar*); **13** (*Mysore*); **14** (*Mandya*); **15** (*Godhra*); **16** (*Anand*); **17** (*Lakhawali*); **18** (*Sukher*); **19** (*Samastipur*); **20** (*Amberi*).

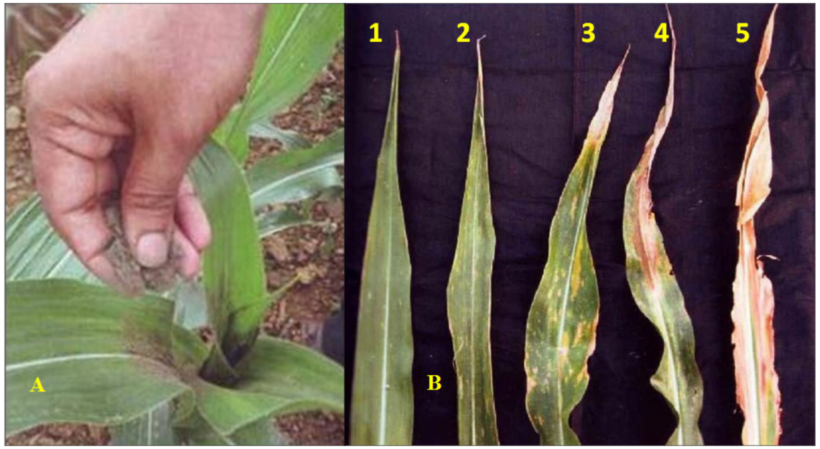


**Figure S2. A.** Inoculation of fungal isolates to the leaf whorl in test plants, *Zea mays* at 7-leaf stage to examine the symptoms and pathogenicity; **B.** Disease rating scale of Payak and Sharma [59].

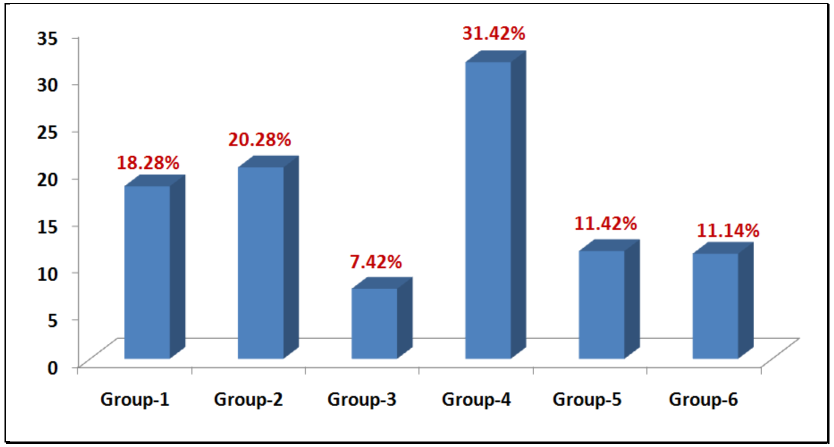


**Figure S3.** Distribution of morphological and pathogenic variability in the total population of fungal species sampled from six maize production zones of India.
